# Injury-induced activation of the endocannabinoid system promotes axon regeneration

**DOI:** 10.1016/j.isci.2023.106814

**Published:** 2023-05-05

**Authors:** Sara Martinez-Torres, Francina Mesquida-Veny, José Antonio Del Rio, Arnau Hervera

**Affiliations:** 1 Molecular and Cellular Neurobiotechnology, Institute for Bioengineering of Catalonia (IBEC), Barcelona, Spain; 2 Department of Cell Biology, Physiology and Immunology, University of Barcelona, Barcelona, Spain; 3 Network Centre of Biomedical Research of Neurodegenerative Diseases (CIBERNED), Institute of Health Carlos III, Ministry of Economy and Competitiveness, Madrid, Spain; 4 Institute of Neuroscience, University of Barcelona, Barcelona, Spain; 5 Clinical Neuroimmunology Group, Vall Hebron Research Institute (VHIR), Barcelona, Spain; 6 Multiple Sclerosis Centre of Catalonia (CEM-CAT), Barcelona, Spain

**Keywords:** Drugs, Molecular medicine, Clinical neuroscience

## Abstract

Regeneration after a peripheral nerve injury still remains a challenge, due to the limited regenerative potential of axons after injury. While the endocannabinoid system (ECS) has been widely studied for its neuroprotective and analgesic effects, its role in axonal regeneration and during the conditioning lesion remains unexplored. In this study, we observed that a peripheral nerve injury induces axonal regeneration through an increase in the endocannabinoid tone. We also enhanced the regenerative capacity of dorsal root ganglia (DRG) neurons through the inhibition of endocannabinoid degradative enzyme MAGL or a CB1R agonist. Our results suggest that the ECS, via CB1R and PI3K-pAkt pathway activation, plays an important role in promoting the intrinsic regenerative capacity of sensory neurons after injury.

## Highlights

• Nerve injury induces activation of the ECS
• MAGL inhibition enhances neurite outgrowth
• CB1R agonist improves intrinsic regenerative capacity of sensory neurons
• Injury-activated ECS promotes neuron regrowth via PI3K-pAkt

## Graphical abstract

**Figure:**
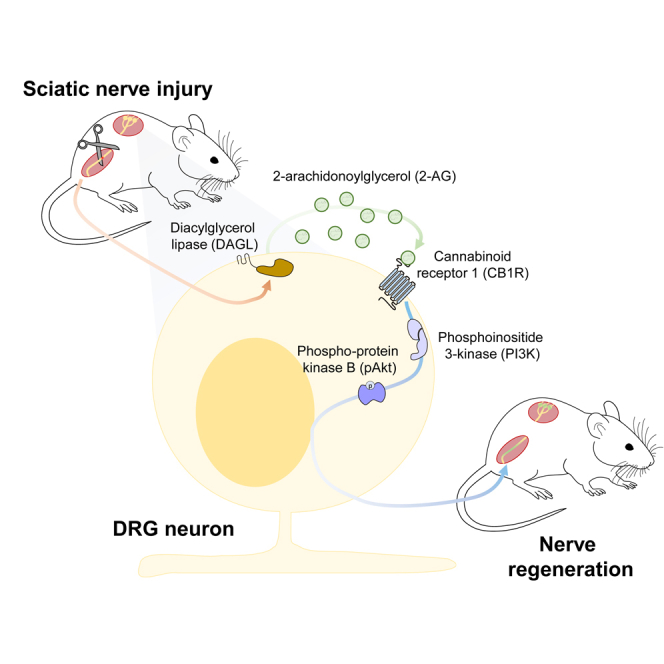

## Introduction

Peripheral nerve regeneration is limited after injury, mainly due to the large distance the axons have to grow in the limbs.^1^^,^^2^ However, the regenerative potential of sensory dorsal root ganglia (DRG) neurons after a peripheral nerve injury or a spinal cord injury (SCI), is enhanced by a prior injury of their peripheral axon, a remarkable ability called the conditioning lesion.^3^^,^^4^ This conditioning lesion paradigm has been thoroughly used as a model to identify multiple regenerative signaling pathways and molecules affecting the intrinsic ability of neurons to regenerate after injury.^5^ Interestingly, new pathways are discovered every year using this paradigm.^6^^,^^7^^,^^8^^,^^9^ While the endocannabinoid system (ECS) has been widely studied for its neuroprotective and analgesic effects,^10^ especially in the context of neuropathic pain,^11^ little is known about its putative role in axonal regeneration, and during the conditioning lesion.

The ECS is an important neuromodulatory system highly expressed in the nervous system. It is composed by cannabinoid receptors, the most important being cannabinoid receptor type 1 (CB1R), highly expressed in neurons, and cannabinoid receptor type 2 (CB2R), predominantly expressed in immune cells. Exogenous and endogenous ligands (endocannabinoids) act on this system by binding to these receptors. The most abundant endocannabinoids in the nervous system are 2-arachidonoylglycerol (2-AG) and arachidonoylethanolamine (AEA or anandamide). Endocannabinoid levels are regulated in an activity-dependent manner by specific synthesis and degradation enzymes. N-acylphosphatidylethanolamine phospholipase D (NAPE-PLD) is the main responsible for the synthesis of AEA, and diacylglycerol lipase (DAGL) is the responsible for the synthesis of 2-AG.^10^ In contrast, monoacylglycerol lipase (MAGL) is predominantly responsible for the degradation of 2-AG,^12^ whereas fatty acid amide hydrolase (FAAH) is responsible for the degradation of AEA.^13^

After nerve injury in mouse models of neuropathic pain, convincing evidence exists regarding increases in local endocannabinoid levels.^14^^,^^15^ This increase is rapidly counteracted by swift cellular uptake and subsequent degradation.^16^ In fact, increasing the endocannabinoid levels by inhibiting their degrading enzymes has been previously used as a therapeutic strategy to increase their analgesic effects.^17^ This approach has profited from a local activation of cannabinoid receptors at sites with high endocannabinoid turnover, rather than global activation of CB1R, which can result in side effects.^16^

In the present study we observed that increasing the endocannabinoid tone through the inhibition of the endocannabinoid degradative enzyme MAGL, increases neurite outgrowth in DRG neurons *in vitro*, via neuronal CB1R activation. Additionally, the CB1R agonist ACEA also enhances neurite outgrowth in cultured DRG neurons, via activation of the PI3K signaling pathway. The administration of this agonist also improves axon growth *in vivo* in mice after sciatic nerve crush. Interestingly, we found that increased neurite outgrowth induced by a conditioning sciatic nerve lesion was blocked by rimonabant administration, a CB1R antagonist, suggesting that CB1R is involved in the conditioning injury-dependent axonal regeneration. Altogether, we have shown the importance of the ECS in promoting the intrinsic regenerative capacity of sensory neurons after injury, opening new venues for the treatment of axonal injuries.

## Results

### Sciatic nerve-conditioning injury promotes axonal outgrowth through 2-AG synthesis

**Figure 1:**
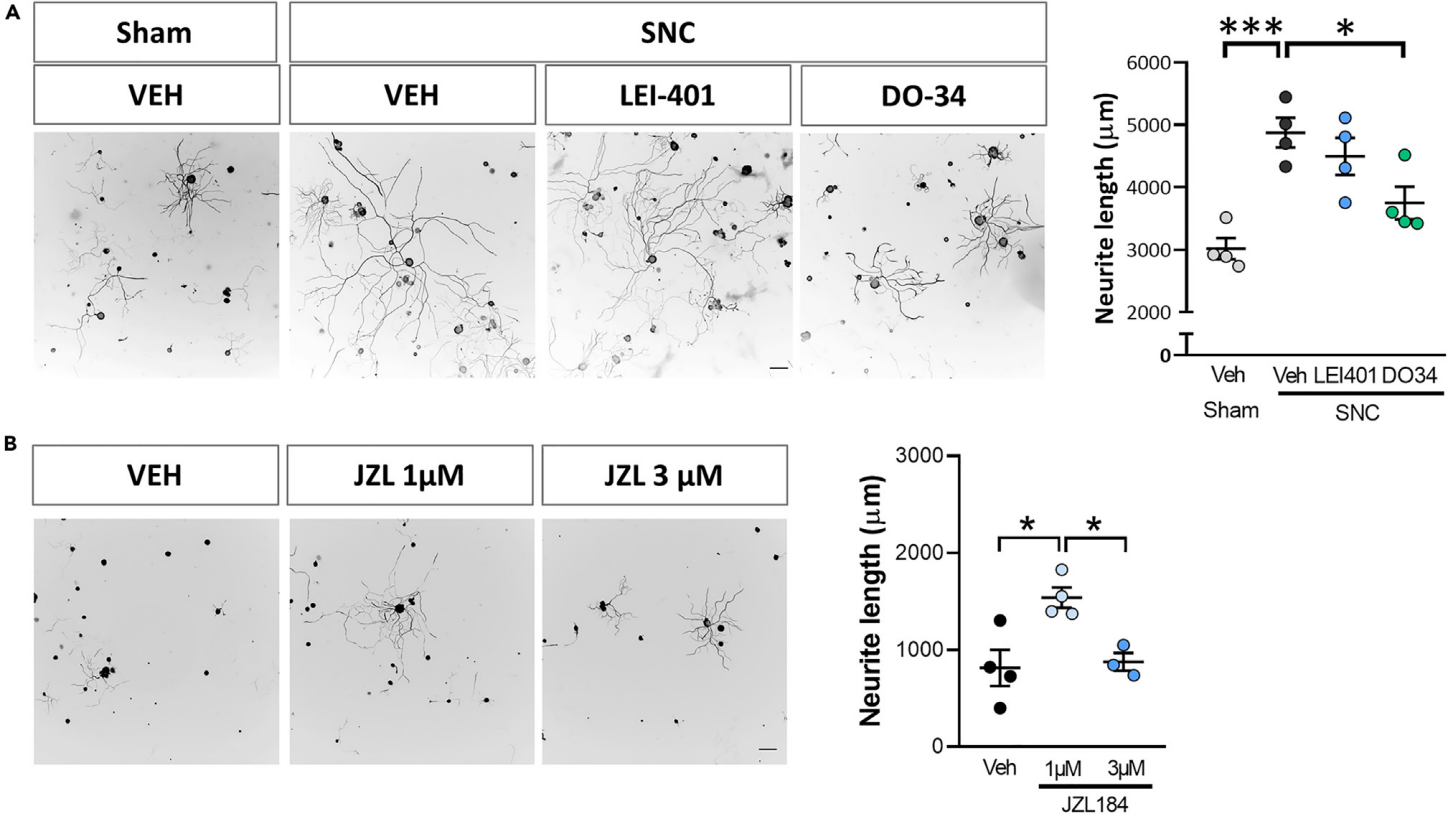
Endocannabinoid signaling promotes injury-induced neurite outgrowth (A) Representative images and graphs of *ex vivo* DRG neurite outgrowth (Tuj-1 positive cells) after *in vivo* sciatic nerve administration of 2-AG synthesis inhibitor (DO34), AEA synthesis inhibitor (LEI401) or vehicle (VEH) before sham surgery or sciatic nerve crush lesion (SNC) (n = 4 cultures from different nerves per group) (B) Representative images and graphs of *in vitro* DRG neurite outgrowth (Tuj-1 positive cells) 24h after vehicle (VEH), 1 μM or 3 μM of MAGL inhibitor (JZL184) administration (n = 3–4 different wells per group). Data are expressed as mean ±S.E.M. ∗p < 0.05 ∗∗∗p < 0.001 denote significant difference after ANOVA followed by Bonferroni post-hoc test. Scale bars 100 μm.

Since the ECS is known to respond upon damage to DRG neurons, we first sought to test if injury-induced endocannabinoid synthesis would support axonal regeneration, including after sciatic “regenerative” conditioning lesion. We measured axon outgrowth in cultured DRG, after a conditioning sciatic lesion combined with LEI-401, a high affinity NAPE-PLD inhibitor,^18^ DO34, a selective DAGL inhibitor, or vehicle. We found that neurite outgrowth induced by a conditioning lesion was partially blocked by administration of DO34, immediately before crush (Figure 1A). Contrarily, LEI-401 did not affect injury-induced DRG outgrowth. These results indicate that the synthesis of 2-AG is necessary for the conditioning effect.

Next, we examined whether 2-AG from DRG neurons is sufficient for DRG outgrowth. We cultured DRG cells *in vitro* and treated them with the MAGL inhibitor JZL184. We observed that the administration of 1 μM JZL184 was sufficient to enhance neurite outgrowth (Figure 1B), although when administered at a higher concentration (3 μM) did not, likely reflecting unspecific effects at this dose. In line with the results of the blockade of the synthesis of AEA through NAPE-PLD inhibition, the inhibition of AEA hydrolysis via URB597, a selective inhibitor of FAAH, did not produce any changes in DRG *in vitro* neurite outgrowth (Figure S1).

To further understand whether the sciatic nerve injury may alter the expression of 2-AG synthesis and degradation enzymes or their cannabinoid receptors, we measured the expression of *Cnr1*, the gene encoding for CB1R, that was significantly reduced 24h after sciatic nerve injury. *Cnr2* expression, the gene encoding for CB2R was instead significantly increased. Neither *Magl* nor *Dagl* expression were altered 24h after sciatic nerve injury (Figure S2).

Together, these data show that the ECS responds in the DRG after a regenerative nerve lesion and that 2-AG is necessary for axonal outgrowth and regeneration of sensory axons.

### CB1R activation promotes axonal regeneration after sciatic nerve injury

**Figure 2:**
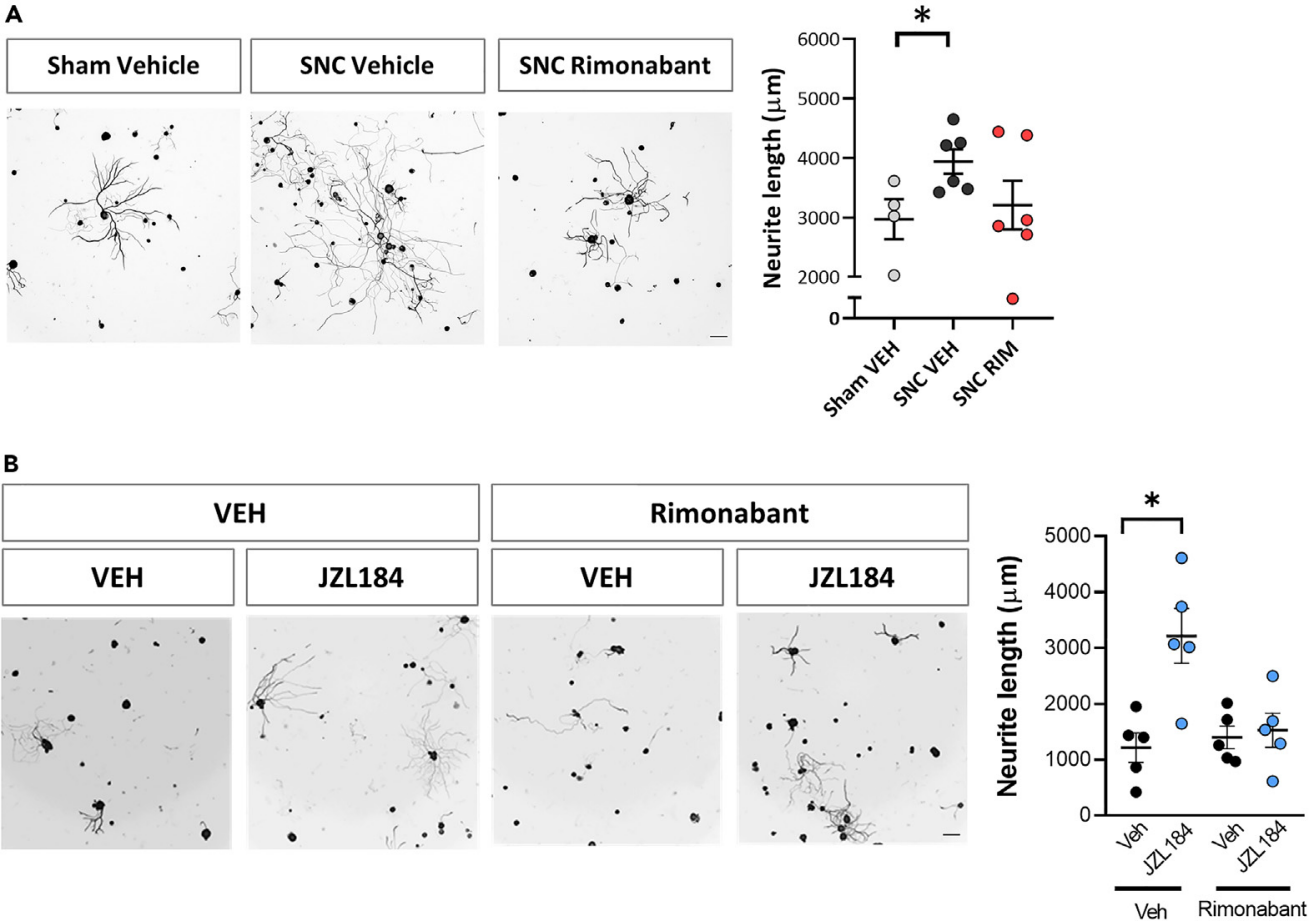
Injury and 2-AG induced outgrowth is induced by CB1R activation (A) Representative images and graphs of *ex vivo* DRG neurite outgrowth (Tuj-1 positive cells) after *in vivo* sciatic nerve administration of the CB1R antagonist rimonabant (RIM) or vehicle (VEH) before sham/sciatic nerve crush (SNC) (n = 4–6 cultures from different nerves per group). (B) Representative images and graphs of *in vitro* DRG neurite outgrowth (Tuj-1 positive cells) 24h after VEH or CB1R antagonist rimonabant (RIM) combined with VEH or JZL184 treatments (n = 5 different wells per group). Data are expressed as mean ± S.E.M. ∗p < 0.05 denotes significant difference after ANOVA followed by Bonferroni post-hoc test. Scale bars 100 μm.

In order to test the effects of CB1R activation after sciatic nerve injury, we measured axon outgrowth in *ex vivo* cultured DRG, 24h after injury combined with rimonabant, a selective CB1R antagonist, or vehicle. Results indicate that neurite outgrowth induced by a conditioning lesion is blocked by administration of rimonabant immediately before crush (Figure 2A).

To test the specificity of the effects induced by JZL184 administration, we combined the administration of the MAGL inhibitor with rimonabant. Interestingly, we found that rimonabant co-administration blocked the JZL184-induced neurite outgrowth (Figure 2B), indicating the CB1R specificity of the effects.

**Figure 3:**
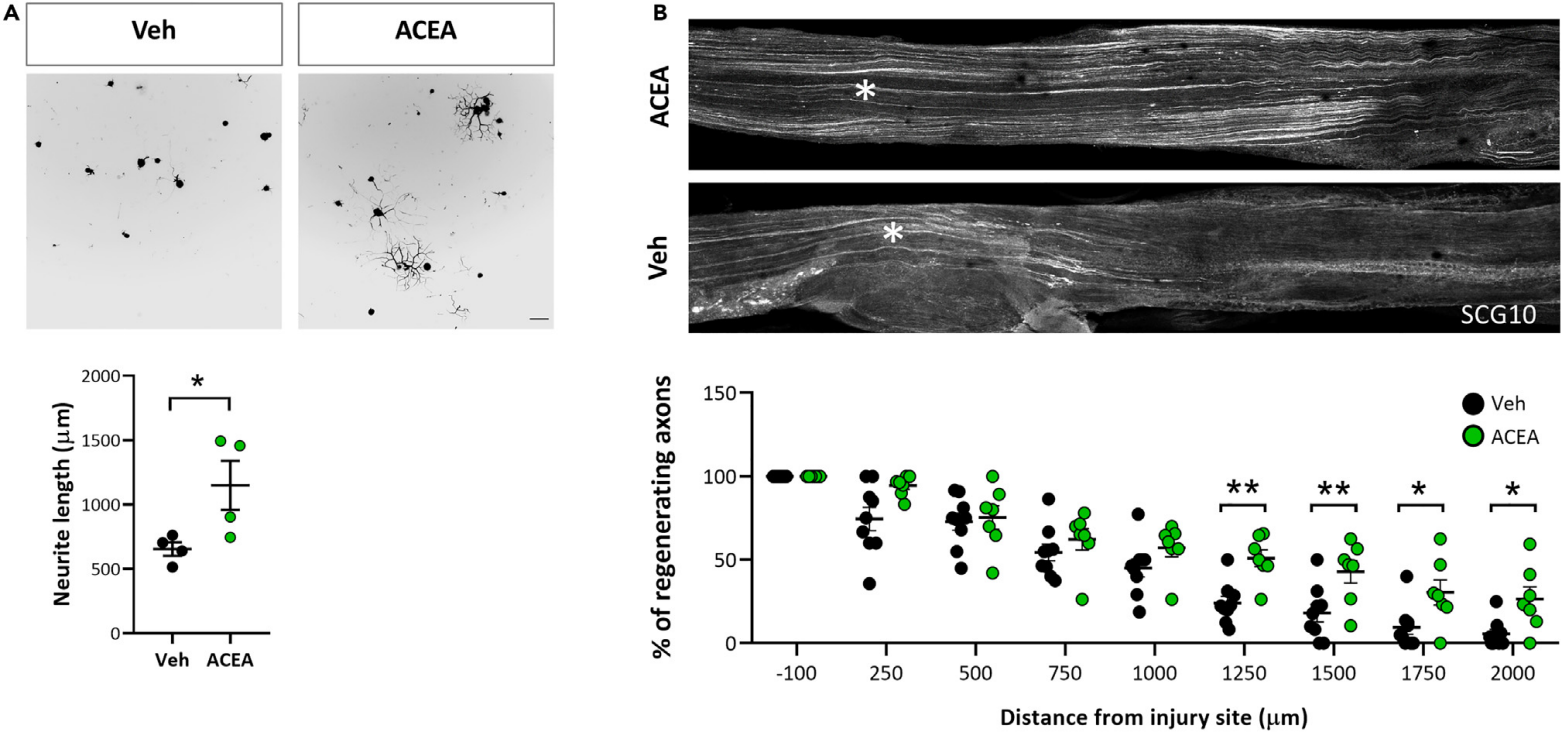
CB1R activation promotes axonal regeneration (A) Representative images and graphs of *in vitro* DRG neurite outgrowth (Tuj-1 positive cells) 24h after vehicle (VEH) or CB1R agonist ACEA administration (n = 4 different wells per group) Scale bar 100μm. (B) Axonal regeneration after SNC measured as number of SCG10-stained axons at indicated distance from injury site 24h after SNC with VEH or ACEA administration. Scale bar 200 μm. Quantification of the percentage of fibers past the crush site normalized to the number of fibers at the crush site plotted as a function of the distance from the crush site (n = 7–9 nerves per group). Data are expressed as mean ± S.E.M. ∗p < 0.05 ∗∗p < 0.01 denote significant difference after ANOVA followed by Bonferroni post-hoc test.

We then sought to phenocopy the regenerative capacity of DRG neurons after sciatic nerve injury, by activating CB1R with ACEA (a highly selective CB1R agonist). We first measured the *in vitro* DRG neurite outgrowth after ACEA administration and found a significant increase in outgrowth compared to vehicle (Figure 3A).

We then asked whether exogenous CB1R activation with ACEA would induce nerve regeneration *in vivo* in the sciatic nerve crush model. We found that ACEA delivered to the sciatic nerve at the time of the crush enhanced the regenerative capacity of injured axons (Figure 3B). These data indicate that CB1R activation is sufficient to promote growth in DRG neurons.

### CB1R activation promotes axonal growth through PI3K-Akt pathway activation

**Figure 4:**
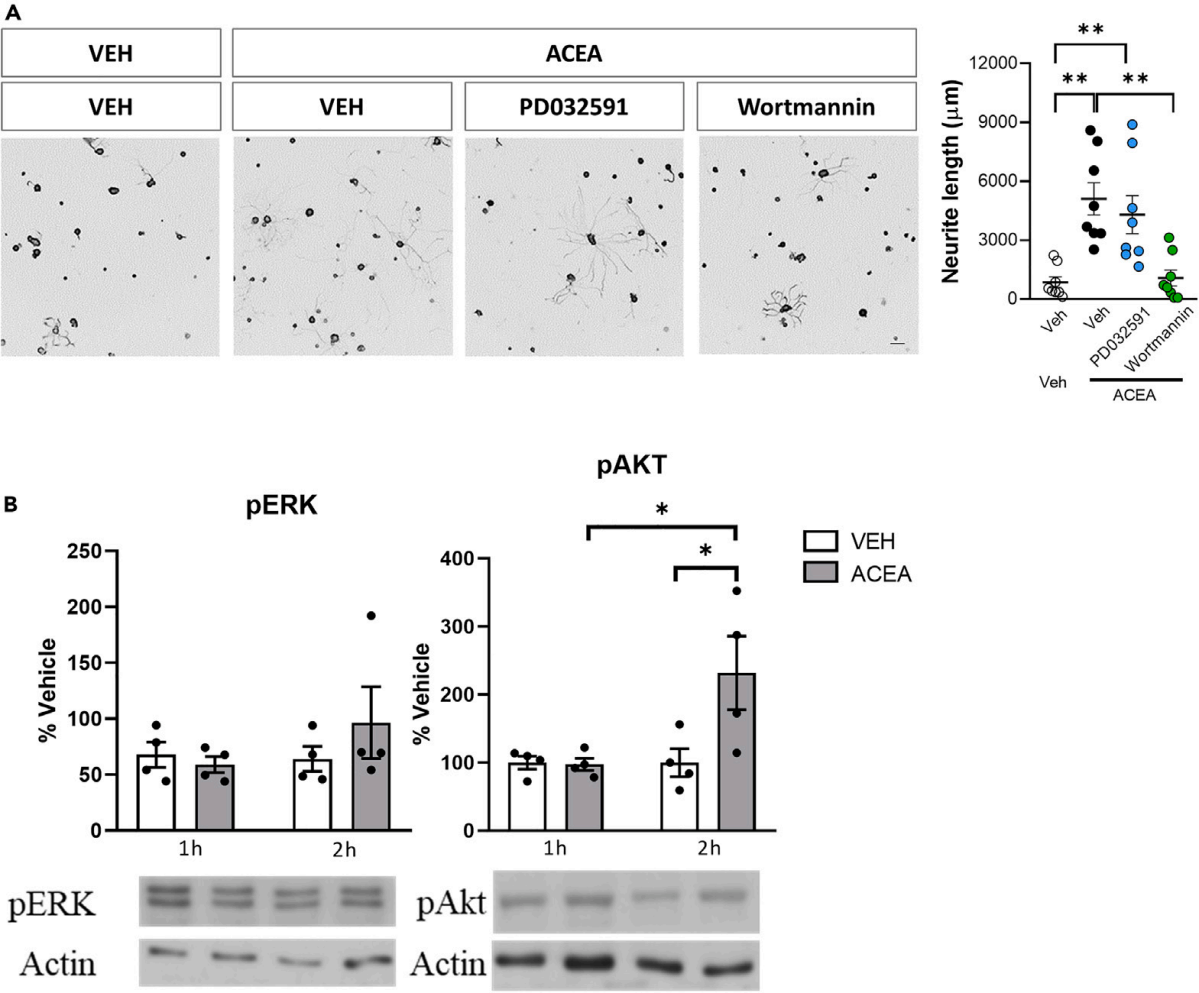
CB1R signaling induces neurite outgrowth through Akt phosphorylation (A) Representative images and graphs of *in vitro* DRG neurite outgrowth (Tuj-1 positive cells) 24h after VEH or ACEA combined with MEK1/2 (PD032591) or PI3K (Wortmannin) inhibitors (n = 8 different wells per group). Data are expressed as mean ± S.E.M. ∗p < 0.05 ∗∗p < 0.01 denote significant difference after ANOVA followed by Bonferroni post-hoc test. Scale bars 100 μm. (B) pERK and pAkt levels in DRG *in vitro* cultures 1 or 2h after ACEA administration measured by Western Blot. Data are expressed as mean ± S.E.M. ∗p < 0.05 denotes significant differences after ANOVA followed by Bonferroni post-hoc test (n = 4 independent cultures).

In order to identify the underlying molecular mechanisms responsible for the CB1R-induced axonal growth, we targeted the two main known downstream actuators of CB1R activation, the MEK pathway and the PI3K pathway, both previously known to be involved in axon growth.^19^^,^^20^ PD032591, an inhibitor of MEK1/2, did not prevent the ACEA-induced outgrowth. Conversely, pharmacological inhibition of PI3K with wortmannin, a selective inhibitor of PI3K, blocked the neurite outgrowth induced by ACEA (Figure 4A), as evidenced by the significant difference versus the Veh-ACEA group and the lack of difference versus the Veh-Veh group. Additionally, ACEA induced an increase in the phosphorylation of Akt 2h after its administration (Figure 4B), but not ERK, proving the role of the PI3K-Akt pathway in the ACEA-CB1R induced neurite growth.

To gain further insight into the downstream molecular pathways supporting CB1R-dependant axonal regeneration, we checked the expression of well-established regeneration associated genes (RAGs), documented to be upregulated after conditioning injury, on DRG neurons 24 h after administration of ACEA (Figure S3), however, we did not observe any change in the expression of any of the analyzed RAGs.

As CB1R activation did not seem to trigger a relevant shift in the expression of RAGs, we tested whether local actin cytoskeleton remodeling was the responsible for the CB1R-dependant induced axonal growth. To this aim, we measured the *in vitro* DRG neurite outgrowth combining the CB1R agonist ACEA with different pharmacological inhibitors of the actin cytoskeleton dynamics: Wiskostatin, an N-WASP inhibitor and C3T, a Rho GTPase inhibitor that stabilizes pre-formed actin filaments and inhibits their disassembly, inhibiting in turn the progression of the growth cone. However, we did not find any of the mentioned inhibitors to affect ACEA-induced outgrowth (Figure S4).

## Discussion

Intrinsic regulatory pathways are a key to axon regeneration post nerve injury. Many studies in the past have identified several pathways, factors, and RAGs involved in the intrinsic control of axon regrowth both in the PNS and the CNS.^5^^,^^21^ While our knowledge in this matter has increased substantially in the last decade, more efforts are still needed to find new therapeutic targets and strategies to treat axonal injuries, both in the PNS and the CNS.

In our study, we identified the ECS as an intrinsic modulator of regeneration. Specifically, we found that this system promotes axon regeneration by activating the CB1R-PI3K-Akt pathway following sciatic nerve injury in mice. The ECS plays an important modulatory part in neurotransmission, affecting different cellular and tissue processes in both homeostatic and pathological settings.^22^^,^^23^^,^^24^^,^^25^

Although we did not observe any change in the expression of the main enzymes responsible for the synthesis and degradation of 2-AG, there seems to be a regulation of their activity, leading to increased 2-AG synthesis, as evidenced by the partial inhibition of the injury-induced neurite growth, when inhibiting DAGL. Additionally, we induced axonal growth *in vitro* by blocking the 2-AG degrading enzyme MAGL. However, the molecular mechanism underlying how a nerve injury induces 2-AG synthesis or DAGL activation remains to be elucidated.

There is strong evidence that the ECS modulates neuronal signaling after injury, a process that has been extensively studied for its analgesic effect during neuropathic pain.^11^^,^^26^ However, in terms of axonal growth, most of the available studies have focused on the role of this system during development of CNS neurons, such as retinal pathways, in which both the activation of CB1R^27^ and CB2R^28^ have been shown to negatively affect neurite growth and induce growth cone collapse during the formation of these pathways. Similarly, CB1R agonists have also shown to cause growth cone collapse in hippocampal GABAergic neurons.^29^ In contrast, but in line with our results, other studies have shown that CB1R antagonists inhibit axonal growth responses^30^ and that pyramidal neurons rely on ECS signaling to initiate the elongation and fasciculation of their long-range axons during development.^31^ The authors of these studies propose that this divergence may be caused by different targeted growth, i.e., cell autonomous guidance versus target-derived cues. Nonetheless, to this date no studies have evaluated the role of the ECS in the regeneration of peripheral sensory axons after injury. Our results revealed that CB1R activation induces growth in proprioceptive neurons and CB1R antagonism blocks the ECS and injury-dependant growth on these neurons.

We also found that nerve injury reduced the expression of CB1R mRNA in the DRG, in accordance with unbiased RNA-Seq data showing a significant reduction in CB1R expression after nerve injury.^32^ Two recent studies have shown different injury-dependant epigenetic mechanisms that control both the downregulation of CB1R expression,^33^ as well as the upregulation of CB2R expression.^34^ A previous study showed that dorsal root ligation injury did not alter the mRNA level of CB1Rs in the injured DRG, however they found a reduction in the percentage of CB1R/IB4 and CB1R/CGRP double-labeled DRG neurons.^35^ This indicates that the apparent overall CB1R expression decrease might be specific to IB4^+^ and CGRP^+^ nociceptors, and the expression on large-diameter proprioceptors might be unaffected.

We also found that the synthetic specific CB1R agonist ACEA promotes axon growth both *in vitro* and *in vivo*. At the same time, pharmacological inhibition of the PI3K-Akt but not the MEK-ERK pathway, the two main downstream mediators of CB1R effects, inhibited ACEA-dependent outgrowth. Additionally, activating CB1R with ACEA leads to the activation of the PI3K-Akt pathway 2h after treatment in cultured DRG neurons, as evidenced by an increased Akt phosphorylation. These results are in line with other studies defining PI3K-Akt as the underlying mechanism of the effects induced by CB1R.^36^^,^^37^^,^^38^ The activation of this pathway in turn promotes the initiation of the regenerative response in DRG neurons as previously reported via other stimuli.^20^^,^^39^^,^^40^ For instance, after nerve injury, Akt is phosphorylated and has been shown to modulate multiple cellular processes affecting axonal growth, including transcriptional and epigenetic alterations, resulting in increased expression of several RAGs,^8^^,^^20^ increased retrograde transport^9^^,^^40^ as well as stimulation of cytoskeleton dynamics.^41^^,^^42^

This CB1R rapid activation of pAkt suggests that endocannabinoids may be part of the early response cascade following a sciatic nerve crush (SNC) and is a key component of the axon regeneration program. Strikingly, the activation of CB1R with ACEA did not affect the expression of the main RAGs known to be upregulated after nerve injury, nor seemed to induce changes in the local actin cytoskeleton.

The present data demonstrate the importance of the ECS in axon growth after injury, while the use of CB1R agonists may represent a novel therapy for nerve injuries. Overall, the present study reveals that the activation of the ECS is associated with axon regeneration and impacts axon regeneration by modulating the activation of PI3K-Akt pathway after nerve injury. This provides deeper insight into the molecular events involved in intrinsic axon regeneration control. Besides the effect of the ECS on PNS nerve regeneration, it would also be interesting to further examine whether the ECS also participates in the axon regeneration process following a CNS injury such as an optic nerve crush or a spinal cord injury.

### Limitations of the study

Currently, there is a lack of techniques to measure endocannabinoid levels in DRG neurons on a single-cell level, which limits our understanding of the cell-specific changes that occur. If new methods become available in the future, these would open the possibility to define how a nerve injury changes the endocannabinoid levels. Additionally, while the mechanisms underlying PI3K-pAkt activation have been extensively studied, the methodology used in this study did not allow for a comprehensive evaluation of gene expression changes or metabolic reprogramming after its activation, and all outcomes analyzed did not show significant changes. Despite this, the PI3K-pAkt pathway has been well established as a key player in promoting axonal regeneration.

## STAR★Methods

### Key resources table

**Table:** 

REAGENT or RESOURCE	SOURCE	IDENTIFIER
**Antibodies**		

Rabbit polyclonal anti-Stathmin-2	Novus	Cat# NBP1-49461, RRID:AB_10011569
Mouse monoclonal anti-Tubulin β 3	Biolegend	Clone: Tuj-1, Cat# 801213, RRID:AB_2728521
Rabbit monoclonal anti-pAkt (Ser473)	Cell Signaling Technology	Clone, D9E, Cat# 4060, RRID:AB_2315049
Rabbit monoclonal anti-Phospho-p44/42 MAPK (Erk1/2) (Thr202/Tyr204)	Cell Signaling Technology	Clone:D13.14.4E, Cat# 4370, RRID:AB_2315112
Mouse monoclonal anti- beta Actin	Thermo Scientific	Clone:AC-15, Cat# AM4302, RRID: AB_2536382

**Experimental models: Organisms/strains**		

Mouse: C57BL6/J Strain	Charles River	Strain Code: 632

**Oligonucleotides**		

Primers	Sigma	CAGATACCACCTTCCGTACCATCAC
		GGTCGACTCCAACGCTATCTTC
		GGAGGAGCCCACATACTTTGCC
		GTGCCTACCTGCTCATGGAATCA
		GGGAGAGAGAGAGCCTTTGC
		GAGGATTTTGCTAACCTGACACC
		GCATTCCTGCTACAGTGCGA
		CCCCTCAACTGTCACTCCAT
		GTGACCCTGTCAGCCACTCT
		AGTCTCCAGGACAGCAAAGC
		GATGACGATATCGCTGCGCTGGTCG
		GTTGTCTCCTGCCGTCATCTTTTC
		GTAGCGGTCAACAGCGGTTAG
		GCTCACCATGACCTTTGCAGCA
		TGCCTGGCCAGCATGTTCAG
		CTTCGGCCTTCTTGTCTTTG
		TTGACGGTAACTGACTCCAGC
		CACCCTGAAGTGCTCAGACG
		CAGGAGCCCTTGAAGATGAG
		GGTCTCCTTTCCTCCACCTC
		TCGTCAGACCTCTCGAACCT
		GCCTGTGGTACGACCAGAGGCATACAG

### Resource availability

#### Lead contact

Further information and requests for resources and reagents should be directed to and will be fulfilled by the lead contact, Arnau Hervera (arnau.hervera@vhir.org).

#### Materials availability

This study did not generate new unique reagents.

### Experimental model and subject details

#### Animals

Young adult (10-12 weeks old) male and female C57BL6 mice (Charles River) were used for *in vivo* and *in vitro* experiments. Mice were housed in a temperature-controlled environment within a range of 21°C - 23°C with 12-hour light/dark cycles, in which they received food and water *ad libitum*. For all experiments, animals were randomly assigned to experimental groups. All experiments from the study were reviewed and approved by Ethics Committee on Animal Experimentation (CEEA) of the University of Barcelona and the local government (Real Decreto 53/2013) and conducted in accordance to European Union (Directive 2010/63/EU) guidelines for the care and use of laboratory animals.

#### Primary cultures

Primary DRG neurons were obtained from freshly dissected DRG from adult (10-12 weeks old) male and female mice. Dissociated cells were plated on poly-D-lysine / laminin coated culture multiwell plates and kept at 37°C in a 5% CO_2_ atmosphere.

### Method details

#### Drugs

To treat DRG culture neurons Rimonabant 1μM (Axon Medchem) and ACEA 7,56nM (Tocris Bioscience) were dissolved in ethanol. JZL184 (Abcam) 1μM and 3μM, URB597 (Sigma- Aldrich) 1μM and 3μM, Wortmannin (Sigma- Aldrich) 1μM, PD032591(Sigma- Aldrich) 1μM and Wiskostatin (Calbiochem) 1μM were dissolved in DMSO. C3T (Cytoskeleton, Inc.) 10ng/μl was dissolved in water.

For *in vivo* administration*,* LEI401 (Tocris Bioscience) 14μg/2ul and DO34 4μg/2ul (Sigma- Aldrich) were dissolved in DMSO; ACEA 1μg/μl (Tocris Bioscience) and rimonabant 1μg/μl (Axon Medchem) were dissolved in ethanol and injected directly in the sciatic nerve.

#### Sciatic nerve regeneration

Briefly, the biceps femoris and the gluteus superficialis were separated by blunt dissection, and sciatic nerve was exposed, immobilized and crushed using fine forceps 2x10s orthogonally. The required compounds were injected locally into the sciatic nerve 5mm rostrally to the crush site. 24h later sciatic nerves were dissected and fixed in 4% paraformaldehyde in PBS at 4°C for 2h, or else, the DRGs were collected and processed for RNA extraction. Whole nerves were immunostained for SCG10/Stathmin-2 (Rabbit, Novus) a marker for regenerating axons.^43^ Number of regenerating axons caudal to the axotomy and their distance from the lesion epicenter were analyzed for both nerves per animal with a confocal fluorescence Zeiss microscope (LSC800).

#### Dorsal root ganglia (DRG) culture

Adult mice were sacrificed and their DRGs were dissected and collected in ice-cold Hank’s balanced salt solution (HBSS) (ThermoFisher Scientific). The DRGs were then enzymatically digested for 45min at 37°C with a solution containing 5mg/ml Dispase II (Merck) and 2.5mg/ml Collagenase Type II (ThermoFisher Scientific), in DMEM (ThermoFisher Scientific), centrifuged, and the digestion solution was then exchanged for DMEM:F12 (ThermoFisher Scientific) media supplemented with 10% heat inactivated FBS and 1x B27 (ThermoFisher Scientific). The DRGs were then dissociated by pipetting and the resulting cell suspension was spun down, resuspended in culture media (DMEM:F-12 media supplemented with 1x B27 and penicillin/streptomycin (P/S) (ThermoFisher Scientific)) and seeded in 48-well plates (3000-4000 cells/well) or 24-well plates (10.000 cells/well, for protein quantification experiments) previously coated with 0.1mg/ml poly-D-lysine (2h, 37°C; Merck) and 2μg/ml laminin (over-night (O/N), RT (room temperature); ThermoFisher Scientific). Cells were kept at 37°C in a 5% CO_2_ atmosphere.

When necessary, compounds were added 2h after plating while for combinatorial experiments, cells were treated with pharmacological inhibitors at 1,5h after plating and ACEA, JZL184 or the corresponding vehicle were added 30 minutes later. All compounds were left in the media for the whole duration of the experiment. Cells were fixed 24h after the treatment.

#### *Ex vivo* DRG culture

Pharmacological treatments were injected locally in sciatic bilateral nerves right before crush or sham. Twenty-four hours later, L4-L6 bilateral DRGs were dissected, dissociated, and plated as above described.

#### Immunocytochemistry

Cells were fixed with ice-cold 4% paraformaldehyde (PFA) for 15min and washed in PBS (0.1M). Blocking solution (PBS-0.25% Triton X-100+ 1% BSA (bovine serum albumin)) was then added and incubated for 1h at RT and then primary antibody (anti-βIII tubulin (Tuj1, 1:1000, BioLegend)), was incubated O/N at 4°C in the same solution. After several washes, Alexa Fluor 568-conjugated secondary antibody was incubated in blocking solution for 1h at RT.

#### Neurite length analysis

Tuj-1 immunocytochemistry was performed and imaged at 10X magnification using an Olympus microscope IX71 with an Orca Flash v.4 (3 images per well). The mean neurite length of large-diameter (>35μm) Tuj-1 positive neurons was manually and blindly quantified with the Neuron J plugin for ImageJ.^44^ Average neurite length per each neuron was quantified by manually tracing each neurite on that neuron, and adding up the total length per neuron. Four to six neurons were quantified per image, and 3 images were quantified per each well or biological replicate. Average arborization or number of nodes per neuron was also quantified, but not included in the results as no differences were observed.

#### Western Blot

1 or 2 hours after the treatment media was aspirated and the cells were lysed in 40μl of sample buffer [0.25M Tris HCL (Sigma-Aldrich), 4%SDS (Sigma-Aldrich), 40% Glycerol (Sigma-Aldrich)] + 10% of β-mercaptoethanol (ThermoFisher Scientific) and detached using a scraper. Cell lysates were then heated at 95°C for 10 min and 10 μl were loaded in SDS-10% polyacrylamide (Bio-Rad) gel electrophoresis (PAGE) gels. The gels were transferred to nitrocellulose membranes (Merck) for 1h at 4°C and the membranes were blocked for 1h with 3% BSA-TTBS at RT and O/N at 4°C with primary antibodies (pAkt (1:1000, Cell Signalling); pERK (1:1000, Cell Signalling)) or 2h at 37°C [β-actin (1:10.000, ThermoFisher Scientific)]. After several washes, Horseradish peroxidase (HRP)-linked secondary antibodies (Dako) were added to the membranes and incubated for 1h at RT, and finally developed with ECL™ substrate (Merck). Intensity quantifications were performed using ImageJ.

#### RNA extraction and reverse transcription

Isolation of total RNA from dorsal root ganglion tissues was performed using a RNeasy Mini Kit (QIAGEN) according to the manufacturer’s instructions. Total RNA concentration was measured using a NanoDrop spectrophotometer. Reverse transcription was performed with 800ng of total RNA from each animal to produce cDNA in a 20 μl reaction using the SuperScript II Reverse Transcriptase (Invitrogen) according to the manufacturer's instructions.

#### Quantitative real-time PCR analysis

Real-time PCR was carried out in a 10 μl reaction using SYBR Green PCR Master Mix (Roche) according to the manufacturer's protocol with a StepOne™ (Applied Biosystems). Specific primers for mouse were used (Table: Primer sequences used).

**Table:** Table: Primer sequences used.

Gene	Forward	Reverse
*Cnr1*	CAGATACCACCTTCCGTACCATCAC	GTTGTCTCCTGCCGTCATCTTTTC
*Cnr2*	GGTCGACTCCAACGCTATCTTC	GTAGCGGTCAACAGCGGTTAG
*Dagla*	GGAGGAGCCCACATACTTTGCC	GCTCACCATGACCTTTGCAGCA
*Mgll*	GTGCCTACCTGCTCATGGAATCA	TGCCTGGCCAGCATGTTCAG
*Cap23*	GGGAGAGAGAGAGCCTTTGC	CTTCGGCCTTCTTGTCTTTG
*Atf3*	GAGGATTTTGCTAACCTGACACC	TTGACGGTAACTGACTCCAGC
*Gdnf*	GCATTCCTGCTACAGTGCGA	CACCCTGAAGTGCTCAGACG
*Sprr1*	CCCCTCAACTGTCACTCCAT	CAGGAGCCCTTGAAGATGAG
*Galanin*	GTGACCCTGTCAGCCACTCT	GGTCTCCTTTCCTCCACCTC
*Bdnf*	AGTCTCCAGGACAGCAAAGC	TCGTCAGACCTCTCGAACCT
*Actin*	GATGACGATATCGCTGCGCTGGTCG	GCCTGTGGTACGACCAGAGGCATACAG

Quantification was performed by using the comparative CT Method (ΔΔCT Method). All the samples were tested in triplicate and the relative expression values were normalized to the expression value of β-actin. The fold change was calculated using the Equation 2^(–ΔΔCt)^ formula, as previously reported.^45^

### Quantification and statistical analysis

Statistics and graphical representation were carried out using Prism 8.1 (GraphPad™ Software). Shapiro-Wilk test was used to verify normality of the distributions. ∗ indicate significant differences in ANOVA followed by Bonferroni’s or Student’s t-test. Plotted data represents mean ± S.E.M (standard error of the mean) as stated in figure legends. All statistical tests and definition of the n for each experiment are detailed in each corresponding figure legend. All tests performed were two-sided, and adjustments for multiple comparisons and/or significantly different variances (Fisher’s F) were applied were necessary, and indicated appropriately in the corresponding figure legend. All data analysis was performed blind to the experimental group by two independent experimenters. Unless otherwise stated, sample size was chosen in order to ensure a power of at least 0.8, with a type I error threshold of 0.05, in view of the minimum effect size that was expected.

## Data Availability

• All data reported in this paper will be shared by the lead contact upon request. • This paper does not report original code. • Any additional information required to reanalyze the data reported in this paper is available from the lead contact upon request.
