# Supplementary material for: Injury-induced activation of the endocannabinoid system promotes axon regeneration
Source: iScience. 2023 May 5;26(6):106814. doi: 10.1016/j.isci.2023.106814 (PMC10205787; doi:10.1016/j.isci.2023.106814)
Supplement: Document S1. Figures S1–S4 [file mmc1.pdf]

iScience, Volume *26*

Supplemental information

Injury-induced activation of the endocannabinoid system promotes axon regeneration

Sara Martinez-Torres, Francina Mesquida-Veny, José Antonio Del Rio, and Arnau Hervera

Supplementary Fig 1.

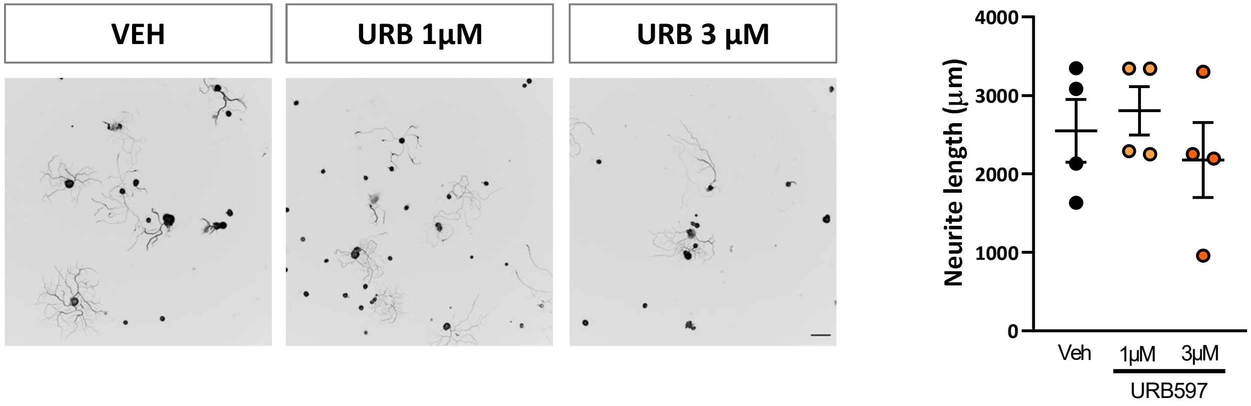

Supplementary Figure 1. FAAH inhibition does not affect neurite outgrowth. Related to Figure 1. Representative images and graphs of *in vitro* DRG neurite outgrowth (Tuj-1 positive cells) 24h after vehicle (VEH), 1µM or 3µM of FAAH inhibitor (URB597) (n = 4 different wells per group). Data are expressed as mean±S.E.M.. Scale bars 100µm.

Supplementary Fig 2.

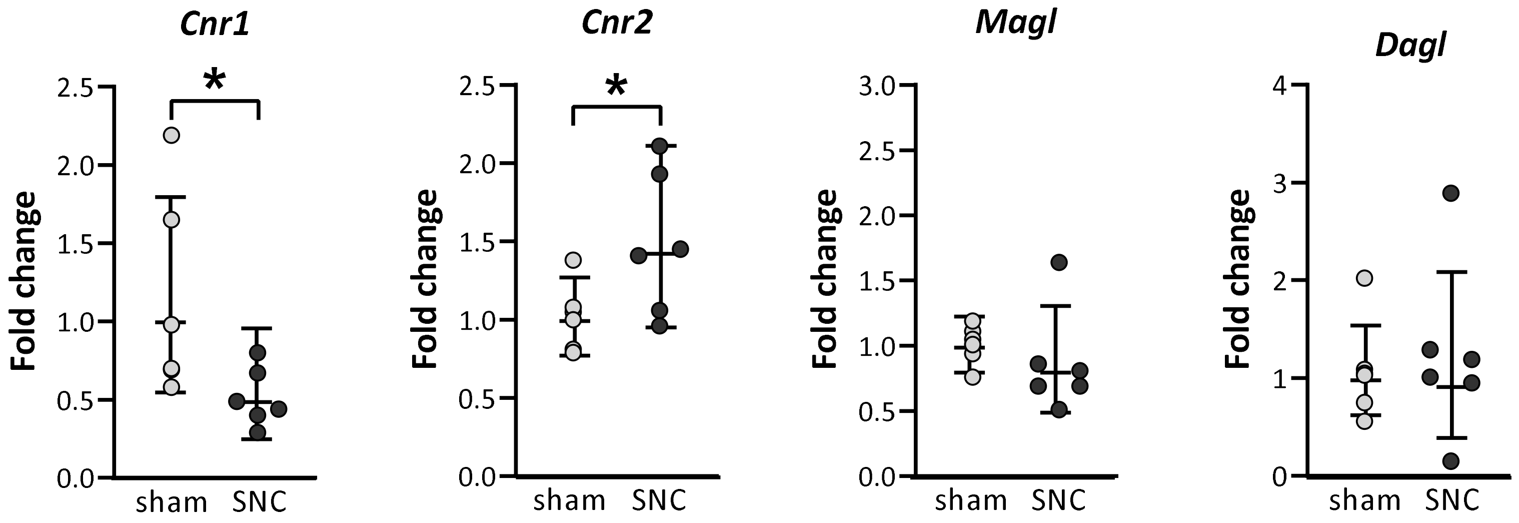

Supplementary Figure 2. Sciatic nerve injury changes cannabinoid receptor expression but not 2-AG synthesis enzymes. Related to Figure 1. DRG mRNA levels of *Cnr1*, *Cnr2*, *Magl* and *Dagl* 24h after sham or sciatic nerve crush (SNC). Data are expressed as $2^{-\Delta\Delta Ct}$ ±upper ($2^{-(\Delta\Delta Ct+SD)}$)/lower ($2^{-(\Delta\Delta Ct-SD)}$) limits. *$p< 0.05$ denotes significant difference between ΔΔCts after Student's T test. (n = 5-6 animals per group).

Supplementary Fig 3.

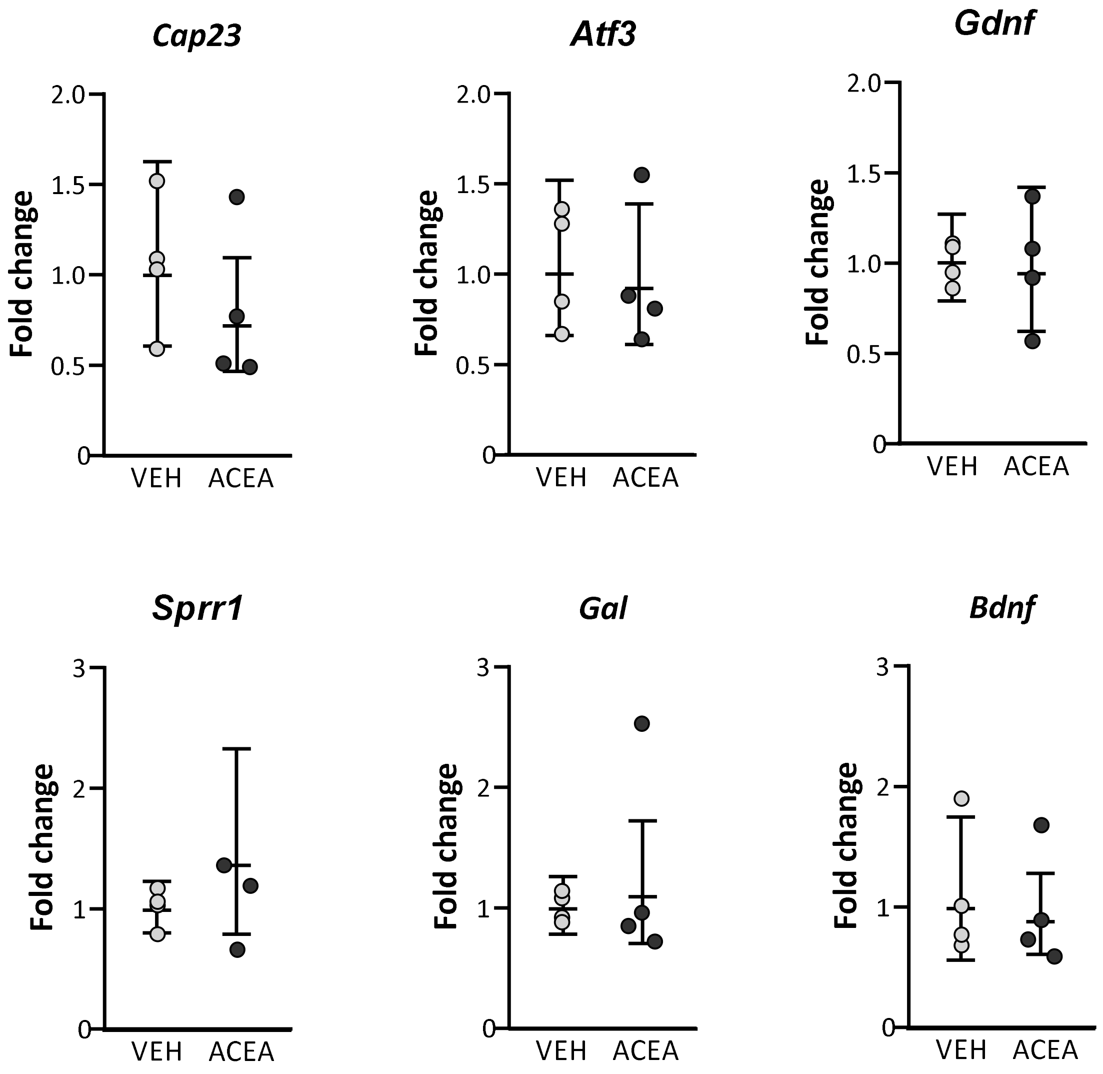

Supplementary Figure 3. ACEA does not induce increase in RAG expression. Related to Figure 4. DRG neuron mRNA levels of RAGS: *Cap23, Atf3, Gdnf, Sprr1, Gal* and *Bdnf* after *in vitro* vehicle (VEH) or ACEA administration. Data are expressed as $2^{-\Delta\Delta Ct}$±upper ($2^{-(\Delta\Delta Ct+SD)}$)/lower ($2^{-(\Delta\Delta Ct-SD)}$) limits. (n= 3-4 biological replicates).

Supplementary Fig 4.

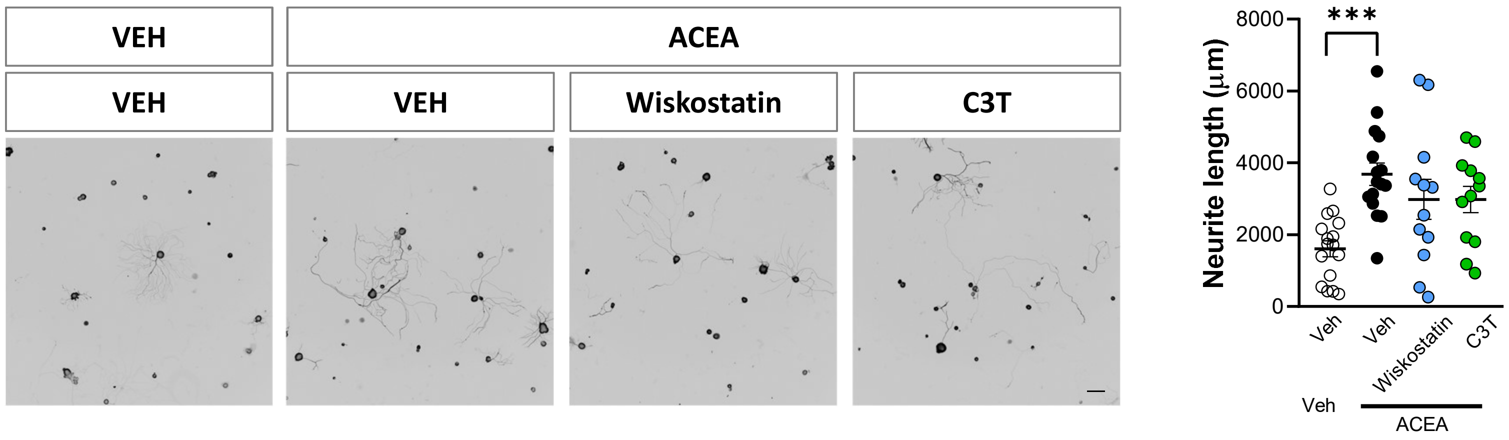

Supplementary Figure 4. ACEA-induced outgrowth is not affected by actin cytoskeleton dynamics inhibition. Related to Figure 4. Representative images and graphs of *in vitro* DRG neurite outgrowth (Tuj-1 positive cells) 24h after VEH or ACEA combined with n-WASP (Wiskostatin) or RhoA (C3T) inhibitors. Data are expressed as mean±S.E.M. ***$p<0.001$ denote significant difference after ANOVA followed by Bonferroni post-hoc test. (n = 12 different wells per group) Scale bars 100μm.
